# The epidemiology of inflammatory skin disease in older adults

**DOI:** 10.1016/j.jdin.2024.09.013

**Published:** 2024-11-08

**Authors:** Danielle West, Alyssa M. Roberts, Benjamin Stroebel, Katrina Abuabara

**Affiliations:** a School of Public Health, University of California Berkeley, Berkeley, California; b Department of Geriatric Medicine, John A. Burns School of Medicine, University of Hawaii at Manoa, Honolulu, Hawaii; c Department of Dermatology, University of California, San Francisco, California; d Department of Physiological Nursing, University of California San Francisco, San Francisco, California

**Keywords:** alopecia areata, atopic dermatitis, epidemiology, inflammatory skin disease, psoriasis, vitiligo

*To the Editor:* Inflammatory skin diseases (ISDs), including psoriasis, atopic dermatitis, alopecia areata, and vitiligo, comprise a clinically diverse group of conditions characterized by chronic systemic inflammation for which many new biological and targeted treatments are under development and now available.^1^ Because these conditions require chronic management and many of the new treatments are costly, it is important to understand the epidemiology of ISDs across the lifespan.

Despite comprising one of the most rapidly growing demographics and having unique comorbidities and treatment considerations, older adults remain underrepresented in ISD research.^2^, ^3^, ^4^ An improved understanding of ISD in this population can inform resource allocation to reduce morbidity. This study aimed to determine ISD prevalence and factors associated with ISD among older adults in a nationally representative survey.

Data came from the Health and Retirement Study, an ongoing national annual survey designed to be representative of adults over age 50 in the United States. In 2019, a sub-survey included questions on chronic inflammatory diseases. The mailed survey received an 83% response rate, and 4774 participants completed all study components and were included in this analysis. ISD prevalence was defined as a self-reported physician diagnosis of relevant conditions for which data were available: atopic dermatitis, psoriasis, vitiligo, and/or alopecia areata.

We found that overall, 12.6% of respondents over age 50 reported having ISD. The highest prevalence was among participants aged 50–59 (18.4%), and the lowest was among participants aged 90-99 (13.5%). Atopic dermatitis was the most common across all groups of older adults (Fig 1). In multivariable logistic regression models adjusted for sampling weights and all of the variables shown in Table I, ISD prevalence was higher in females than males (odds ratio 1.58, 95% CI 1.26-1.99), and lower in Black and Hispanic than White respondents (OR 0.71, 95% CI 0.50-1.00 and OR 0.67, 95% CI 0.50 -0.89, respectively). The association with socioeconomic variables was complex: while education and insurance were positively associated with ISD, middle household net worth was associated with a lower risk of ISD than low household net worth in multivariate analyses (OR 0.75 95% CI 0.57-0.99). However, there were no significant univariate associations with household net worth categories. Our findings come from a representative sample of U.S. older adults but are limited by self-report and lack clinical detail.

**Fig 1:**
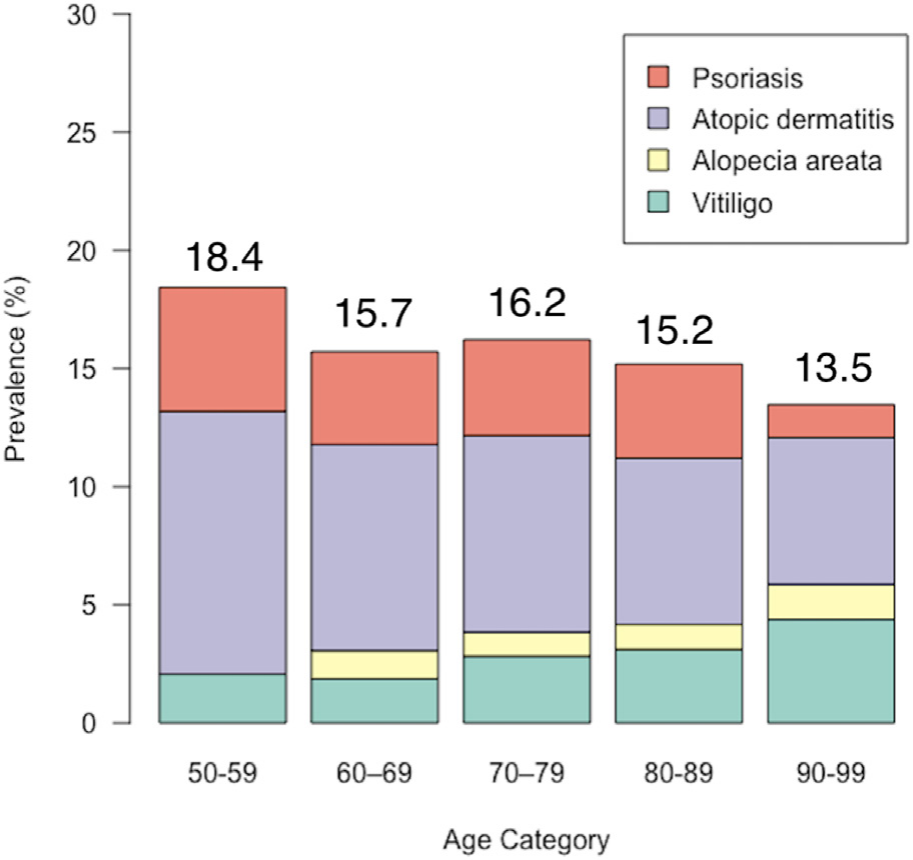
Frequency of self-reported previous or current diagnosis of inflammatory skin disease. Due to small subgroup sample sizes, participants under age 50 (*n* = 29) and above age 100 (*n* = 4) are not included in the illustration.

**Table I:** Respondent characteristics and logistic multivariate associations of characteristics with any inflammatory skin disease

	No ISD	Psoriasis	Atopic dermatitis	Alopecia areata	Vitiligo	Odds of any ISD∗ (95% CI)
Prevalence (%)	3647 (76.4%)	177 (3.7%)	367 (7.7%)	46 (1.0%)	113 (2.4%)	
Age (SD)						
Mean	71.5 (9.51)	70.7 (9.80)	70.0 (9.9)	71.9 (10.3)	73.6 (11.4)	0.99 (0.97-1.00)
Gender (%)						
Male	1521 (41.7)	67 (37.9)	113 (30.8)	8 (17.4)	34 (30.1)	ref
Female	2125 (58.3)	110 (62.2)	254 (69.2)	38 (82.6)	79 (69.9)	**1.58 (1.26-1.99)**
Race/Ethnicity (%)						
Non-Hispanic White	2450 (67.2)	135 (76.3)	274 (74.7)	33 (71.7)	76 (67.3)	ref
Black	620 (17.0)	11 (6.21)	58 (15.8)	10 (21.7)	15 (13.3)	**0.67 (0.50-0.89)**
Hispanic	462 (12.7)	23 (13.0)	23 (6.27)	2 (4.35)	19 (16.8)	**0.71 (0.50-1.00)**
Other	114 (3.13)	8 (4.52)	12 (3.27)	1 (2.17)	3 (2.65)	0.62 (0.30-1.17)
Educational degree (%)						
No degree	479 (13.3)	15 (8.47)	22 (6.01)	3 (6.52)	18 (16.1)	**0.61 (0.43-0.86)**
GED or HS	1890 (52.5)	92 (52.0)	182 (49.7)	25 (54.3)	69 (61.7)	ref
2 or 4 y college	805 (22.4)	47 (26.6)	96 (26.2)	11 (23.9)	15 (13.4)	1.10 (0.82-1.44)
Master or professional	426 (11.8)	23 (13.0)	66 (18.0)	7 (15.2)	10 (8.93)	**1.67 (1.16-2.37)**
Employment status (%)						
Unemployed	2615 (71.8)	125 (70.6)	254 (69.2)	35 (76.1)	96 (85.0)	ref
Employed	1028 (28.2)	52 (29.4)	113 (30.8)	11 (23.9)	17 (15.0)	0.99 (0.75-1.32)
Marital status (%)						
Married	2347 (64.4)	121 (68.4)	233 (63.5)	29 (63.0)	67 (59.3)	ref
Single	1297 (35.6)	56 (31.6)	134 (36.5)	17 (37.0)	46 (40.7)	0.96 (0.77-1.21)
Health insurance (%)						
Uninsured	3 (0.1)	0 (0.00)	0 (0.00)	0 (0.00)	0 (0.00)	ref
Government insurance	5314 (97.6)	266 (97.8)	518 (96.5)	80 (100)	199 (97.1)	**1.57 (1.14-2.18)**
Private insurance	126 (2.31)	6 (2.21)	19 (3.54)	0 (0.00)	6 (2.93)	1.95 (0.76-4.42)
Household net worth (%)						
≤$45,000	866 (31.8)	43 (32.6)	87 (34.0)	11 (37.9)	38 (42.2)	ref
$45,001-211,050	927 (34.1)	38 (28.8)	78 (30.5)	4 (13.8)	28 (31.1)	**0.75 (0.57-0.99)**
≥$211,051	928 (34.1)	51 (38.6)	91 (35.6)	14 (48.3)	24 (26.7)	0.77 (0.58-1.02)

Bolded values denote statistical significance (*P* < .05).

*AA*, Alopecia areata; *AD*, atopic dermatitis; *CI*, confidence interval; *GED*, General Educational Development test; *HS*, high school; *ISD*, inflammatory skin disease; *OR*, odds ratio; *PS*, psoriasis; *ref*, reference; *SD*, standard deviation; *VI*, vitiligo.

∗ Results from multivariable model including all covariates in the table.

In summary, we found that ISDs are common among older adults, impacting 1 in 8 U.S. residents over age 50. While previous studies found that racial minority groups have an increased odds of ISD, our study found the opposite. This could be explained by differential access to dermatologists and residual confounding that may not be fully addressed by adjusting for insurance type in our models.^5^ Given the size of the prevalence of ISDs among the growing older adult population, further inquiry into the burden and impact of ISDs in older adults is important.

## Conflicts of interest

Dr Abuabara is a consultant at Target RWE, Amgen, Nektar therapeutics, and Sanofi, and receives research grants to her institution from Pfizer and Cosmetique Internacional SNC.
